# Potential of Complementary and Alternative Medicine in Preventive Management of Novel H1N1 Flu (Swine Flu) Pandemic: Thwarting Potential Disasters in the Bud

**DOI:** 10.1155/2011/586506

**Published:** 2010-10-13

**Authors:** Rajesh Arora, R. Chawla, Rohit Marwah, P. Arora, R. K. Sharma, Vinod Kaushik, R. Goel, A. Kaur, M. Silambarasan, R. P. Tripathi, J. R. Bhardwaj

**Affiliations:** ^1^Division of Chemical Biological, Radiological and Nuclear (CBRN) Defence, Institute of Nuclear Medicine and Allied Sciences, Defence Research and Development Organization, Brig. S.K. Mazumdar Road, Delhi 110054, India; ^2^Department of Chemistry and Biochemistry, Faculty of Medicine, Trakia University, Armeiska Street 1, 6000 Stara Zagora, Bulgaria; ^3^Centre for Disaster Management Studies, Guru Gobind Singh Indraprastha University, Kashmere Gate, Delhi 110006, India; ^4^Division of Disaster Management, National Disaster Management Authority (NDMA), A1 Safdarjung Enclave, New Delhi 110001, India

## Abstract

The emergence of novel H1N1 has posed a situation that warrants urgent global attention. Though antiviral drugs are available in mainstream medicine for treating symptoms of swine flu, currently there is no preventive medicine available. Even when available, they would be in short supply and ineffective in a pandemic situation, for treating the masses worldwide. Besides the development of drug resistance, emergence of mutant strains of the virus, emergence of a more virulent strain, prohibitive costs of available drugs, time lag between vaccine developments, and mass casualties would pose difficult problems. In view of this, complementary and alternative medicine (CAM) offers a plethora of interesting preventive possibilities in patients. Herbs exhibit a diverse array of biological activities and can be effectively harnessed for managing pandemic flu. Potentially active herbs can serve as effective anti influenza agents. The role of CAM for managing novel H1N1 flu and the mode of action of these botanicals is presented here in an evidence-based approach that can be followed to establish their potential use in the management of influenza pandemics. The complementary and alternative medicine approach deliberated in the paper should also be useful in treating the patients with serious influenza in non pandemic situations.

## 1. Introduction

Viruses are a serious threat to the health of people in all parts of the world. For most bacterial diseases, several effective drugs are available, however, viral diseases are often difficult to treat primarily because viruses spread and mutate very rapidly. The control and treatment of a viral infection depends mainly on the availability of antiviral drugs, which are few and usually are not virucidal but simply prevent replication in the host. It has become imperative to develop effective medical strategies for the management of common viral diseases like influenza, which can assume pandemic proportions and become a major threat to humanity. Complementary and alternative medicines have been used effectively by humans over several centuries for treating various diseases and can be effectively employed to target the host response during influenza outbreaks [1] and are the focus of the present paper.

## 2. Influenza Reassorted Virus: Is a Global Disaster in the Making?

### 2.1. Swine Flu: The Current Pandemic

**Figure 1:**
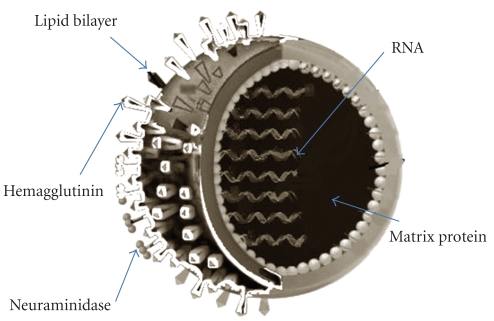
The H1N1 Virus.

The etymology of the word Pandemic can be traced to a Greek word *pandemos “pertaining to all people” *and has been derived from *pan – “*all” + *demos – “*people”. Pandemic is referred to as a pervasive epidemic of contagious disease affecting throughout the whole of a country or one or more continents at the same time. It relates basically to the virus's geographical spread rather than its severity. There have been three flu pandemics in the 20th century and these occurred in 1918, 1957, and 1968. Influenza A viruses are single-stranded RNA viruses with an eight segmented genome and belong to the family Orthomyxoviridae. The viral haemagglutinin (HA) and neuraminidase (NA) proteins envelope glycoproteins (Figure 1) and are the key antigens against which humoral immune responses are directed.

### 2.2. Novel H1N1 Flu

**Figure 2:**
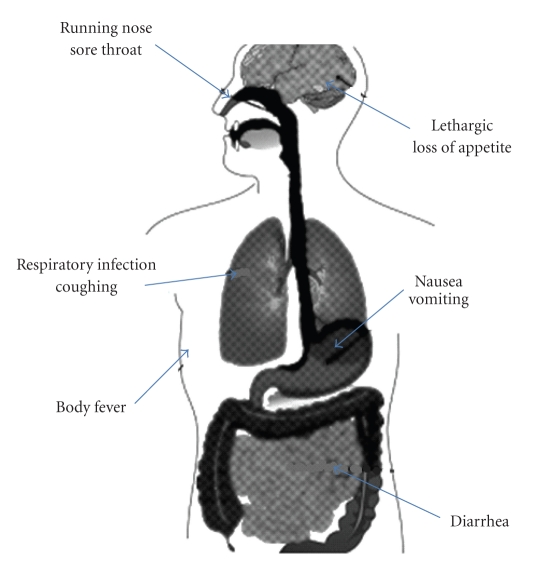
Symptoms of Swine flu due to novel H1N1 viral attack.

Swine flu is a highly contagious respiratory disease of pigs with low mortality (1%–4%), is species-specific in nature, and outbreak usually occurs once in a year with an upsurge in Autumn and Winter in temperate zones. One such virus, namely, Influenza A H1N1 virus has evolved the capacity to cross species barrier (i.e., pig to humans) and has spread widely amongst humans. This led to a pandemic situation covering nearly the entire globe with a significant pace in terms of spread. More than 213 countries and overseas territories or communities have reported laboratory confirmed cases of pandemic influenza H1N1, including at least 16226 deaths. Novel H1N1 flu is characterised with the onset of sudden fever (>38°C/100.4°F), cough, tiredness, headache, chills, loss of appetite aching muscles, paining joints, runny nose, sneezing, and sore throat. In case of acute respiratory infections, shortness of breath is one of the additional symptoms. In addition to these mild complications, diarrhoea, and vomiting are also observed in nearly one out of four patients (Figure 2). 

### 2.3. Novel H1N1 Flu: Impact Assessment, Mitigation, and Control Measures

**Figure 3:**
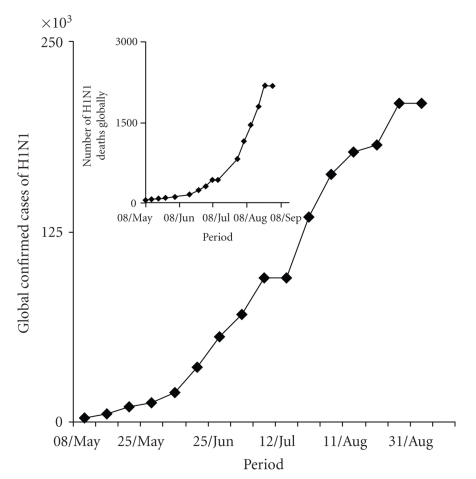
Confirmed cases of H1N1 at global level & number of deaths (April-Sep 2009).

**Figure 4:**
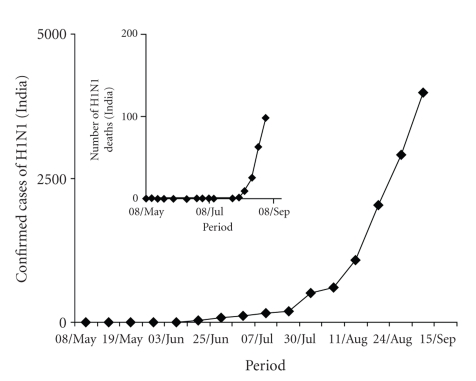
Confirmed cases of H1N1 in India & number of deaths (April-Sep 2009).

There is a significant variation in number of cases among different regions identified by World Health Organisation (WHO). The number of cases as of now followed the trend American region > European > Western Pacific > South East Asian Region. The followup of H1N1 outbreak in April 2009, reaching Phase V and then VI has exhibited a systematic spread from a single source to global arena. By May 2009, it was established and anticipated that the disease will attain pandemic proportions (Figure 3). The entrance of H1N1 virus into the South Asian region with maximal proportion of global population led to a significant increase in number of confirmed cases, with a significant number of global deaths. Till 10 Feb 2010, the number of confirmed human cases of H1N1 in India were 29187 and among these cases, the number of deaths had reached 1278 (Figure 4). The total number of cases has been reported from more than 10 states of the country. A rise in number of cases of H1N1 in the coming months can be expected due to winter approaching. The WHO, however contends that climate does not have any effect. 

A report published in May, 2009 revealed that maximal number of confirmed cases was reported in the age group either <15 years or 15–29 years, though, more number of deaths occurred in older people [2]. Immunocompromised patients or old people with ageing or preexisting disorders are more vulnerable, and therefore complementary and alternative medicine can be used to boost the natural immune system in a holistic manner. In addition, proper preinfection and postinfection planning can also help in controlling the mortality rate due to Swine flu [3, 4]. 

## 3. Modern Therapeutic Interventions: How Safe Are We?

Modern system of medicine is based on a medical model that basically offers symptomatic treatment and focuses more on therapy. It emphasizes on the use of drugs, mechanical testing, invasive treatments like surgery, and a passive approach towards the patient. In modern medicine, two classes of antiviral medication are currently available for the treatment of seasonal human influenza. Neuraminidase inhibitors like oseltamivir (Tamiflu), zanamivir (Relenza), and adamantanes (rimantadine and amantadine). Interestingly, the starting material for the synthesis of Tamiflu is also a herb (*Illicium verum*). Genetic and phenotypic analyses indicate that H1N1 is susceptible to oseltamivir and zanamivir, but resistant to the adamantanes. These drugs mainly target three influenza envelope proteins: hemagglutinin, neuraminidase, and the matrix 2 ion channel protein. A recent study emphasized the urgent need for augmentation of oseltamivir (Tamiflu) stockpiles with additional antiviral drugs, including Zanamivir (Relenza), based on an evaluation of the performance of these drugs in the scenario that the 2009 H1N1 “Swine Flu” neuraminidase (NA) were to acquire the Tamiflu-resistance (His274Tyr) mutation, which is currently wide-spread in seasonal H1N1 strains [5]. Treatment with amantadines may lead to the rapid production of resistant viruses, and over-use of these drugs has probably contributed to the recent spread of resistance [6]. Laboratory studies have also shown that it is possible that even the use of suboptimal doses of these drugs as a prophylactic measure might contribute to the development of drug resistance [7].

## 4. Why Alternatives Are Needed?

**Figure 5:**
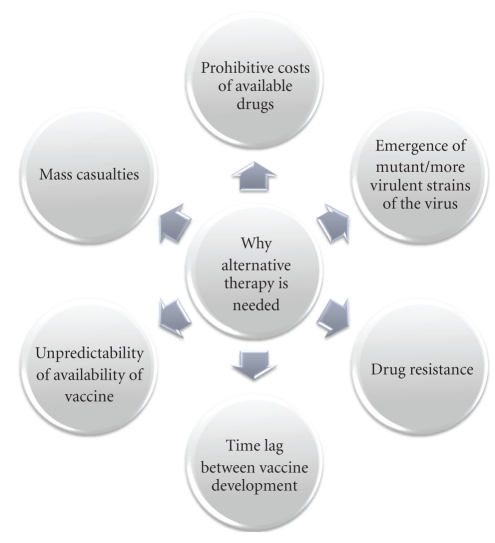
Need for an alternative therapy to manage novel H1N1 viral infection.

The need for alternatives arises because of some of the following reasons (Figure 5).

## 5. Solutions from Nature

### 5.1. Learning from Traditional Wisdom

Complementary and alternative medicine has been utilized for several years in various parts of the world to alleviate human disease. The preventive role of complementary and alternative medicine, in particular, cannot be overemphasized. “Complementary” implies that it joins conventional medicine as an equal counterpart to make the healing process complete. Alternative medicine substitutes for conventional medicine. It neither works with nor enhances conventional medicine, but rather acts as its replacement. Complementary and alternative medicine has been used for the well-being of the general population, especially when conventional modern medicine has failed to deliver and has also been used at times in conjunction with conventional medicine to obtain synergistic effects. 

The traditional Indian System of Medicine, namely, Ayurveda [8], Traditional Chinese medicine (TCM) [8, 9], Japanese traditional medicine (Kampo) [10], Unani [11], Siddha, and so forth, belong to the category of complementary and alternative medicines. For some or the other reason the alternative herbal treatment systems have so far been unable to enter mainstream medicine [12], though serious efforts are being made, in view of their effectiveness, to develop a strong evidence-based standardization of Ayurveda, Siddha, Unani, Traditional Chinese Medical Therapy [13], and other CAM so that they can aptly fit into the modern medicinal framework. 

Ayurveda, the traditional Indian system of medicine, has been widely used since centuries and a number of plants of the Indian subcontinent have been utilized for tackling almost every human ailment. Ayurveda's focus is more on creating an energetic balance at the higher energetic or inner level. It sees all life and nature as constantly evolving towards a higher level of consciousness [14]. Ayurvedic formulations have an impact at this higher level of consciousness, as well as the more gross body level. Ayurveda seeks to connect us with this intelligence inherent in nature. These profound concepts, based upon an astute understanding of the universal laws and practical observations about the world around, give us the indication about the holistic approach of Ayurveda and its potential in alleviating many health-related problems afflicting the whole of humanity. Several antiviral agents have been isolated from plants as a result of chemical and pharmacological studies in the recent years, and many have been derived from leads based on Ayurvedic and other traditional medicine principles. These agents include a variety of polyphenols, flavonoids, saponins, glucosides, and alkaloids [15]. Here, we discuss various potential herbs that have been evaluated for their efficacy against flu viruses and hence can prove to be useful to combat the novel H1N1 pandemic.

#### 5.1.1. Glycyrrhiza glabra

Also known as Yashtimadhu (Sanskrit), Mulathee (Hindi), and Licorice (English), *Glycyrrhiza glabra* (Papilionaceae) derives its flavour principally from a sweet-tasting compound called anethole (“trans”-1-methoxy-4-(prop-1-enyl)benzene). Additional sweetness in licorice comes from glycyrrhizic acid, an antiviral compound significantly sweeter than sugar [16]. Powdered licorice root is an effective expectorant, and has been used for this purpose since ancient times, especially in Ayurvedic medicine. The roots of the plant have been used for throat and upper respiratory tract-related infections and contain many phenolic compounds such as flavonoids and their glycosides, coumarin, and cinnamic acid derivatives. Particularly from the Indian species, Glucosides, Liquiritin, and Isoliquiritin have also been isolated. The active compounds Triterpine, Saponins, particularly Glycyrrhizinic acid have shown antiviral activity [17]. Polysaccharide fractions obtained from *Glycyrrhiza glabra* stimulate macrophages [18] and hence elevate and assist immune stimulation [19]. Also animal studies have revealed its efficacy against the influenza a virus that is mediated by stopping the virus replication [20]. Glycyrrhizic acid present in the plant inhibits virus growth and inactivates virus particles [21]. 

#### 5.1.2. Ocimum sanctum

*O. sanctum*, also known as Tulsi (Hindi) and Holy Basil (English), is an aromatic plant of the family Lamiaceae. The plant, as a whole, is a treasure house of potent compounds with its leaves, seeds, and roots, as well as flower being medicinally important and is considered divine by the Hindus. *O. sanctum* is considered to be an adaptogen par excellence [22, 23]. It harmonizes different processes in the body and is helpful in acclimatizing to stress. The main chemical constituents of *O. sanctum* are oleanolic acid, ursolic acid, rosmarinic acid, eugenol, carvacrol, linalool, and *β*-caryophyllene [23]. The antiviral activity of eugenol has been reported. *Ocimum* extracts are used in ayurvedic remedies for common colds, headaches, stomach disorders, inflammation, heart disease, various forms of poisoning, and malaria. Traditionally, *O. sanctum* is taken in many forms as herbal tea, dried powder, fresh leaf, or mixed with ghee. Essential oil extracted from Karpoora *O. sanctum* is mostly used for medicinal purposes and in herbal cosmetics, and is widely used in skin preparations due to its antimicrobial activity [24]. Recent studies suggest that *O. sanctum* may be a COX-2 inhibitor, like many modern painkillers, due to its high concentration of eugenol (1-hydroxy-2-methoxy-4-allylbenzene) [25]. *O. sanctum* is reported to be an effective treatment for diabetes and high cholesterol [26]. *O. sanctum* also shows promise for protection against radiation damage [27, 28]. *O. sanctum* leaves contain highest percentage of essential oils, infusion of which is given in malaria. Juice of the leaves is taken internally and is very effective in skin diseases such as itches fungal infections. Fresh leaves also cure chronic fever and when mixed with honey and ginger juice, it is useful in cough and bronchitis [29]. During the past decade the plant has been extensively investigated and has been shown to possess a range of biological activities such as antibacterial activity [30], antifungal activity [31], and antiviral activity [32]. Nitric oxide production was induced by *O. tenuiflorum* extracts in stimulated peripheral blood mononuclear cells *in vitro* and the active component(s) responsible for immunomodulatory action were identified [33]. The extract was also used to stimulate the cells individually and in combination with mitogens as well [34]. The antimicrobial properties of *O. sanctum* make it useful for the management of novel H1N1 flu. 

#### 5.1.3. Alium sativum

*Alium sativum*, also known as Lahsan (Hindi) and Garlic (English), belongs to family Alliaceae. *A. sativum* has been used throughout recorded chronicles for both culinary and medicinal purposes. It has a characteristic pungent, spicy flavor. *A. sativum* has been used for hundreds of years to treat fungal, parasitic, and viral infections, and has anti-inflammatory properties that show promise for prevention of cardiovascular disease. It is known to kill influenza virus *in vitro* [35]. Researchers are focusing on an extract of *A. sativum* called ajoene, which appears to protect CD+ cells from attack by HIV early in the viral life cycle. At low concentrations, the drug appears to have little toxicity, and its anti-HIV activity is 45 times more powerful than the drug dextran sulfate. Ajoene is found only in fresh *A. sativum* and is not readily procurable. Recent investigations reveal that *A. sativum* impairs the activity of the liver enzymes that process protease inhibitors and raises the protease inhibitor levels. The *in vitro* antiviral activity of *A. sativum* extract (GE) on human cytomegalovirus (HCMV) was also evaluated in tissue cultures, plaque reduction, and early antigen assay. A dose-dependent inhibitory effect of GE was evident when GE was applied simultaneously with HCMV [36]. The *in vitro* antiviral effect of garlic against parainfluenza virus type 3 and human Rhinovirus type 2 has also been evaluated [37].

#### 5.1.4. Cocos nucifera

The coconut (*Cocos nucifera*) belongs to the Family Arecaceae (palm family). The most common form of its usage is the coconut oil, which is extracted from the kernel of matured coconut (*Cocos nucifera*). Throughout the tropical regions, it has been the primary source of fat in the diets of millions of people since aeons. *Cocos nucifera* oil has a long history of use, both as food and as medicine, throughout the world. It holds a high place of respect in Ayurvedic medicine in India. In folk remedies around the world, coconut is used to treat a wide assortment of ailments including abscesses, alopecia, amenorrhea, asthma, blenorrhagia, bronchitis, bruises, burns, cachexia, calculus, colds, constipation, cough, debility, dropsy, dysentery, dysmenorrhea, earache, erysipelas, fever, flu, gingivitis, gonorrhea, hematemesis, hemoptysis, jaundice, menorrhagia, nausea, phthisis, pregnancy, rash, scabies, scurvy, sore throat, stomachache, swelling, syphilis, toothache, tuberculosis, tumors, typhoid, venereal diseases, and wounds [20]. It has been reported that certain fatty acids, primarily medium-chain fatty acids (MCFA), and their derivatives (e.g., monoglycerides) have potent antiviral properties [59]. When *C. nucifera* oil is consumed, the medium-chain triglycerides (MCTs) are broken down into individual medium chain fatty acids and monoglycerides, which can kill or inactivate pathogenic microorganisms inside the body. The antiviral action, attributed to monolaurin (the monoglyceride of lauric acid), is that of solubilizing the lipids and phospholipids in the envelope of the pathogenic organisms causing the disintegration of their outer membrane. There is also evidence that MCFA interfere with the organism's signal transduction [60] and the antimicrobial effect in viruses is due to interference with virus assembly and viral maturation [61].

#### 5.1.5. Zingiber officinale

*Zingiber officinale* (Ginger) is a plant which belongs to the family Zingiberaceae. The characteristic odor and flavor of ginger root is caused by a mixture of zingerone, shogaols, and gingerols, volatile oils that comprise of about one to three percent of the weight of fresh ginger. In laboratory animals, the gingerols increase the motility of the gastrointestinal tract and have analgesic, sedative, antipyretic, and antibacterial properties [62]. Ginger contains gingerol, a pungent ingredient of ginger volatile oil with sulphur-containing compounds (allicin, alliin, and ajoene), and enzymes (allinase, peroxidase, and myrosinase). The antibiotic properties of allicin are well known. The allicins have fibrinolytic activity, which reduces platelet aggregation by inhibiting prostaglandin E2. Compounds in ginger also increase levels of antioxidant enzymes, including superoxide dismutase and glutathione peroxidase, which may be beneficial in inflammatory reactions triggered by viral infections [63]. Anti-influenza agents have been isolated from *Z. officinale*. TNF-*α*, reported as anti-influenza cytokine, has been reported to be present in ginger [64].

#### 5.1.6. Phyllanthus emblica

The Indian gooseberry (*Phyllanthus emblica*, syn. *Emblica officinalis*) is a deciduous tree of the Euphorbiaceae family. It is also known as Amlaka (sanskrit) and Amla (Hindi). In traditional Indian medicine, dried and fresh fruits of the plant are used. All parts of the plant, including the fruit, seed, leaves, root, bark, and flowers, are used in various Ayurvedic/Unani Medicine herbal preparations. According to Ayurveda, *Emblica officinalis* fruit is sour and astringent in taste, with sweet, bitter, and pungent secondary tastes. 

Methanol extract of the fruit of *Emblica officinalis* has potent inhibitory action against human immunodeficiency virus-1 reverse transcriptase. *Emblica officinalis* aqueous extracts are used in Cuban traditional medicine for their antiviral activity against Hepatitis B virus and A and B influenza virus. The cytotoxicity of the extract was tested by means of colony-forming ability and growth-inhibition assays, as well as by measuring the mitotic index. Apoptosis induction and cell-cycle kinetics were analyzed by cytofluorimetric methods [72]. In Ayurvedic polyherbal formulations, *Emblica officinalis* is a common constituent, and most notably is the primary ingredient in an ancient herbal preparation called Chyawanprash [73], which is itself an effective adaptogen and immunity booster that could help control swine flu infection. 

#### Tinospora cordifolia

*Tinospora cordifolia*, also called Guduchi, is a herbaceous vine of the family Menispermaceae indigenous to the tropical areas of India, Myanmar, and Sri Lanka. The active constituents are diterpene compounds, including tinosporone, tinosporic acid, cordifolisides A to E, syringen, the yellow alkaloid, berberine, Giloin, crude Giloininand, and a glucosidal bitter principle, as well as polysaccharides, including arabinogalactan polysaccharide (TSP) [74, 75]. These compounds possess adaptogenic and immunomodulating properties. Picrotene and bergenin, possessing antioxidant properties have been reported from *Tinospora*. *Tinospora cordifolia *has been studied extensively for its immunomodulating activities. The active principles of *Tinospora cordifolia* were found to possess immunomodulatory activities and caused significant increases in IgG antibodies in serum, along with macrophage activation [76]. Enhancement in humoral immunity, evidenced by the hemagglutination titre, along with stimulation of cell-mediated immunity were observed in the leukocyte migration inhibition tests [77]. The plant has immense potential for use against novel H1N1 flu since it is a potent immunostimulant. 

#### 5.1.8. Mentha piperita

*Mentha piperita*, family Labiatae, is a herbaceous rhizomatous perennial plant widely used in Ayurveda [78]. It contains about 1.2%–1.5% essential oil. The volatile oil, also known as menthae piperitae aetheroleum, contains 30–70% free menthol, menthol esters and more than 40 other compounds. The principal components of the oil are menthol (29%), menthone (20%–30%), and menthyl acetate (3%–10%). Pharmaceutical grade oil, produced by distilling the fresh aerial parts of the plant at the beginning of the flowering cycle, is standardized to contain no less than 44% menthol, 15%–30% menthone, and 5% esters, in addition to various terpenoids. Other compounds found in it are flavonoids (12%), polymerized polyphenols (19%), carotenes, tocopherols, betaine, and choline [79]. The antimicrobial and antiviral activity of menthol has been reported. *Mentha piperita* has significant antiviral activity [80]. Menthol is virucidal against influenza, herpes, and other viruses *in vitro*. Aqueous extracts of peppermint leaves exhibited antiviral activity against Influenza A, Newcastle disease virus, Herpes simplex virus, and Vaccinia virus in egg and cell-culture systems [81]. The oil contains terpenoids such as *α*-pinene or *β*-pinene, *α*-phellandren, and also ester-connected with menthol or free acetic acid and isovaleric acid, which are mainly responsible for the antimicrobial activity of the herb [82].

#### 5.1.9. Azadirachta indica

*Azadirachta indica* (Neem in Hindi) is a tree in the mahogany family Meliaceae. Three bitter compounds that have been extracted from neem oil are nimbin, nimbinin, and nimbidin, respectively [83]. The seeds contain a complex secondary metabolite azadirachtin. All parts of the plant yield *β*-sitosterol. The antiviral activity of azadirachtin, nimbin, and nimbidin has been reported. *Azadirachta indica *extracts possess antidiabetic, antibacterial, and antiviral properties. The tree stem, root, and bark possess astringent and tonic properties [84, 85]. *In vitro* antiviral activity of aqueous neem leaves extract, assessed in cloned cells of larvae of *Aedes albopictus* cells employing virus inhibition assay, showed inhibition in a dose-dependent manner [86].

*Azadirachta indica *has traditionally been used as an antiviral, and animal and laboratory research has shown promising results. While researchers have still not pinpointed the exact mode of action of neem phytoconstituents, there is some evidence to show that they interfere with viral reproduction, thus minimizing the impact of viral infections. The effect of *A. indica *leaf extract and pure compound (Azadirachtin) on the replication of Dengue virus type-2 has also been reported. Thus, neem can serve as a source of promising future antiviral drugs [85].

#### 5.1.10. Aegle marmelos

*Aegle marmelos* also called Bael (Hindi) belongs to family Rutaceae. It contains primarily alkaloids, coumarins, and steroids. The leaves contain skimianinc, sterol, and aegelin. The active constituent of the fruit is marmorosin, which is identical to imperatorin. Coumarins contained in the fruits are altoimperatorin and *β* sitosterol. Roots of the tree have been found to contain psoralin, xanthotoxin, scopoletin, and tebamide.* A. marmelos *from India is reported to possess imperetorin [87], which has certain interesting biological properties such as analgesic, anti-inflammatory, antibacterial, and antiviral properties. 

All parts of this tree-stem, bark, root, leaves, and fruit at all stages of maturity have been used in Ayurveda since ages. Medicated oil prepared from bael leaves gives relief from recurrent colds and respiratory infections. Its regular use builds up resistance to colds and coughs. The unripe fruit possesses significant antiviral activity. 

#### 5.1.11. Trachyspermum ammi

*Trachyspermum ammi,* called as Ajwain in Hindi and Bishops weed in English, is a member of the family Apiaceae. The principal constituents of the essential oil from the fruit are the phenols, mainly thymol and some carvacrol. The oil possesses p-cymene, g-terpinene, *α*- and *β*-pinenes, and dipentene, minute amounts of camphene, myrcene, and carene [88]. The essential oil is a strong antiseptic [89], antispasmodic, aromatic, bitter, diaphoretic, digestive, diuretic, expectorant, and tonic [90]. It is used internally in the treatment of colds, coughs, influenza, and asthma. The essential oil is also added to various cough medicines as well [89]. 

#### 5.1.12. Andrographis paniculata

*Andrographis paniculata* (Kalmegha in Hindi) is a herbaceous plant in the family Acanthaceae, native to India and Sri Lanka. It is sometimes called “Indian Echinacea” because it is believed to provide much the same benefits as Echinacea. Andrographolide, the major constituent of the extract is implicated towards its pharmacological activity. Studies have been conducted on the cellular processes and targets modulated by andrographolide treatment of immune cells. *Andrographis* was found to both reduce the symptoms and shorten the duration of colds in clinical trials [90]. *Andrographis paniculata* also reduced the cold symptoms such as fatigue, sore throat, sore muscles, runny nose, headache, and lymph node swelling [91]. Unlike the Echinacea, *Andrographis* does not have any side effects. 

#### 5.1.13. *Terminalia chebula* Retz

*Terminalia chebula*, is a deciduous tree of family Combretaceae native to Southern Asia from India and Nepal east to Southwestern China (Yunnan), and south to Sri Lanka, Malaysia, and Vietnam. It is regarded as a universal panacea. The dry nut's peel from this plant is used to cure cold-related nagging coughs. The bark/peel of the nut is placed in the cheek and this generates a huge amount of saliva as the material does not dissolve. The resulting saliva, bitter in taste, is believed to have medicinal qualities to cure cold related coughs. Its fruits possess digestive, anti-inflammatory, anthelmentic, cardiotonic, aphrodisiac, and restorative properties and are additionally beneficial in cough and colds. *Terminalia chebula* is an important medicine, which often promotes health through successive steps of purification and detoxification. It is known to have strong antimutagenic activity, because of its very rich content vitamin C [92]. Also it is an established potent free radical scavenger [93]. 

## 6. Traditional Chinese Medicine

Some plants that are extensively used in Traditional Chinese Medicine and could prove useful for the management of swine flu are as follows.

### 6.1. Sophora flavescens

*Sophora flavescens* is a species of plant in the genus Sophora. Its roots are regionally called ku shen or kushenin [94] which is the source of flavonoids and is used as traditional Chinese medicine [95]. Its roots also contain quinolizidine alkaloids, including matrine and its oxide [96], that interfere TNF-alpha and IL-6, suggesting that oxymatrine may inhibit the expression of the above proinflammatory cytokines [97]. Recent studies have shown that the plant also contains 8-Prenylkaempferol (8-PK), a prenylflavonoid in its roots. The principle bioactive constituents of *S*. *flavescens* are the major quinolizidine alkaloids matrine (MT) and oxymatrine (OMT), which were reported to exhibit sedative, depressant, antitumor, antipyretic, and cardiotonic activities.

Due to its antiviral action, the plant has been the focus of attention for innovative studies. The recent studies to appraise its efficacy against H1N1 infection have yielded positive outcomes. Cell inoculation with H1N1 evoked a significant induction in RANTES accumulation accompanied with time-related increase in nuclear translocation of nuclear factor-*κ*B (NF-*κ*B) and interferon regulatory factor 3 (IRF-3), but showed no effect on c-Jun phosphorylation. 8-P*κ* could significantly inhibit not only RANTES production, but also NF-kappaB and IRF-3 nuclear translocation [98].

### 6.2. Toddalia asiatica

*Toddalia asiatica* (Lopez root) belongs to family Rutaceae, a woody liana, found in mainly Philippines and southern China. It is a large, spiny, woody vine, which is pungent in all its parts and possesses sharp recurved prickles. The leaves are trifoliolate. The leaflets are stalkless, ovate-elliptic, 3 to 8 centimeters long, 5 to 15 millimeters wide, rounded at the base, and pointed at the apex. The flowers are small, greenish-white, 5 millimeters across, and borne on terminal cymes or from the upper leaf axils. The fruit is small, nearly spherical, less than 1 centimeter in diameter, borne in fairly large clusters, 3 to 5 grooved, and with as many cells, and orange red when ripe. The seed is solitary in each cell. *T. asiatica* is used traditionally in the treatment of malaria, sprains, cough, fever, neuralgia, epilepsy, dyspepsia, and other disease conditions. Extracts of the plant have been reported to have anticancer, antimicrobial, antiviral (anti-HIV), and antifeedant activities. A wide range of chemical constituents such as benzophenanthridine alkaloids, coumarins, cyclohexylamides, and terpenoids have been isolated especially from the root bark of the plant [99]. The essential oil from the plant is a highly potent antimicrobial agent [100, 101].

### 6.3. *Schefflera heptaphylla*, Chinese Herbal Tea

*Schefflera heptaphylla* belongs to the family Scarabaeoidea. Leaves are palmately compound, rarely unifoliolate (not in China); margins entire to serrate; stipules united within petiole. Inflorescence is a terminal or pseudolateral panicle or compound raceme; flowers arranged in umbels, heads, or racemes; bracts pubescent, deciduous, or persistent. Pedicels are not articulate below ovary. Calyx rim is entire or 5-toothed. Petals are arranged 5-1, d are valvate. It is a critically endangered species. It is polyphyletic [102–104]. 

*Frodin is the principal ingredient of a herbal tea formulation that is widely used for the treatment of common cold in Southern China. An extract of the long leafstalk of the compound leaf of *S. heptaphylla* exhibited the most potent antiviral activity against respiratory syncytial virus. Triterpenoids, namely, 3alpha-hydroxylup-20[29]-ene-23,28-dioic acid and 3-epi-betulinic acid 3-O-sulfate, together with an inactive saponin, 3alpha-hydroxylup-20(29)-ene-23,28-dioic acid 28-O-alpha-l-rhamnopyranosyl-(1-->4)-O-beta-d-glucopyranosyl-(1-->6)-beta-d-glucopyranoside are present in the plant [105]. Three caffeoylquinic acid derivatives, namely 3,4-di-O-caffeoylquinic acid, 3,5-di-O-caffeoylquinic acid, and 3-O-caffeoylquinic acid, were also isolated from this plant. These compounds were tested for their activity against Respiratory Syncytial virus. Studies revealed that they exerted their anti-RSV effects via the inhibition of virus-cell fusion in the early stage, and the inhibition of cell-cell fusion at the end of the RSV replication cycle [106].

### 6.4. *Camellia sinensis* or Green Tea

Green tea is a type of tea made solely from the leaves of *Camellia sinensis* that has undergone minimal oxidation during processing. Green tea originated in China and has now become associated with many cultures in Asia from Japan to the Middle East. Recently, it has become more widespread in the West, where black tea is traditionally consumed. 

Green tea is particularly rich in polyphenolic compounds and catechins. Catechin derivatives have shown pronounced antiviral activity, observed for derivatives carrying moderate chain length (7–9 carbons). The derivatives exerted inhibitory effects for all six influenza subtypes tested including three major types of currently circulating human influenza viruses (A/H1N1, A/H3N2, and B type), H2N2 and H9N2 avian influenza virus. The compounds strongly inhibited adsorption of the viruses on red blood cell (RBC) [107]. 

The disease preventive properties of green tea are mainly due to the presence of polyphenols like epigallocatechin-3-gallate (EGCG), epicatechin, epicatechin-3-gallate, and epigallocatechin (EGC). These polyphenols comprise about one-third of the weight of the dried leaf of the plant. These catechins have been reported to possess diverse pharmacological properties, including antioxidative, anti-inflammatory, anticarcinogenic, antimutagenic and antimicrobial effects. Green tea has the ability to enhance humoral and cell-mediated immunity and is, therefore, useful for preventing influenza by inhibiting flu replication, using potentially direct virucidal effect [108].

### 6.5. Panax quinquefolius

*Panax quinquefolius*, commonly known as American Ginseng, is an herbaceous perennial in the ivy family that is commonly used in medicine. It is native to Eastern North America, though it also cultivated beyond its range in places such as China [109]. American ginseng contains dammarane-type ginsenosides as the major biologically active constituents. Dammarane type ginsenosides include two classifications: the 20(S)-protopanaxadiol (ppd) and 20(S)-protopanaxatriol (ppt) classifications. American ginseng contains high levels of Rb1, Rd (ppd classification), and Re (ppt classification) ginsenosides that are helpful in prevention of common cold [110]. In Eastern Europe, ginseng is widely used to improve overall immunity to illness. It appears that regular use of ginseng may prevent colds. Studies have been done on *Panax* to reveal that they effectively provide immunity to individuals against influenza [111]. 

### 6.6. Echinacea

Echinacea is a genus of nine species of herbaceous plants in the family Asteraceae. It is purely native to North America. The principal effect of these species is to stimulate the body immune system. It appears to activate the body's infection-fighting capacity, which is primarily due to the presence of various chemical compounds such as polysaccharides, phenols, alkylamides, and Cichoric acid. Studies in Europe have concluded that Echinacea, when taken at first sign of cold, reduced cold symptoms or shortened their duration [112]. There is some evidence that, when taken at the onset of a cold or flu, Echinacea can help patients recover faster and reduce their symptoms while they are sick. For example, Echinacea significantly reduced symptoms such as headache, lethargy, cough, and aching limbs [113, 114] when administered to people with flu-like illnesses. The constituents found in Echinacea were found to increase antibody production, raise white blood cell counts, and stimulate the activity of key white blood cells [115–118].

Some of the studies have also reported about the allergic effects of *Echinacea. *Echinacea should not be taken by persons with progressive systemic and autoimmune disorders, connective tissue disorders, or related diseases. It should not be used with immunosuppressants or hepatotoxic drugs, [119] and has the potential to interfere with anesthesia.

## 7. CAM for the Management of H1N1: The Way Ahead

**Figure 6:**
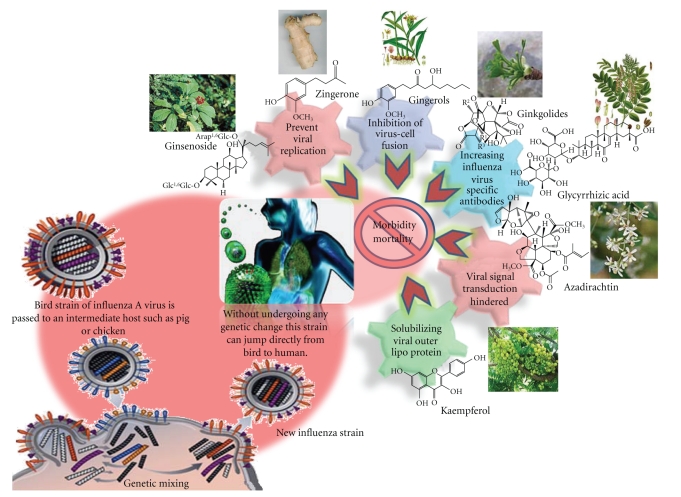
The mechanism of action of some isolated compounds from herbs and their role in prevention and treatment of novel H1N1 flu.

From the foregoing account it is apparent that several medicinal plants have the potential to be used effectively for the management of H1N1. These medicinal herbs mainly act *via *two basic approaches against H1N1 infection, namely, enhancement of overall immunity of the individual or by acting against the virus by preventing viral replication or by inhibiting viral signal transduction.  Table 1 enumerates the various medicinal herbs from all over the globe that have been found to be potent against the virus, while Figure 6 aptly depicts that genetic modifications have led to the evolution of the deadly H1N1 virus and discusses the mechanistic aspects *vis-a-vis* isolated phytomolecules. The herbs that boost the overall immunity of the body by stimulating specific or nonspecific entities of the immune system, thereby improving the individual's ability to tackle infections, have been listed in Table 2.

Antiviral agents have been isolated from plants as a result of a recent resurgence of chemical and pharmacological studies. These agents include a variety of polyphenols, flavonoids, saponins, glucosides, and alkaloids [15]. Such isolated compounds may prove to be highly beneficial in controlling the mortality/morbidity rate resulting due to H1N1 swine flu since they target various pathways and act at multifarious levels. The mechanisms by which some of the potent bioactive compounds act have been elucidated to some extent. For example kaempferol, isolated from *Emblica officinalis*, acts *via* solubilisation of viral outer lipoprotein [72]; azadirachtin, isolated from *Azadirachta indica*, hinders the viral transduction [86]; glycyrrhizic acid from *Glycyrrhiza glabra* [17] and ginkgolides from *Ginkgo biloba *increase virus-specific antibodies in the infected individual. Zingerone and gingerols from *Zingiber officinalis *prevent viral replication and also intervene in the process of virus-host fusion [63]. 

The need of the hour is not only to isolate and characterize compounds from the various medicinal plants but to utilize medium, high and ultrahigh-throughput methods of screening, molecular profiling, which will involve genomic and proteomic approaches and nanotechnology-based antiviral drug development. It is anticipated that rapid advances in the field of bioinformatics would lead in the future to improvisation of antiviral effectiveness of phytocompounds, *via* virtual antiviral screening of phytocompounds, for effective management of H1N1 flu.

**Figure 7:**
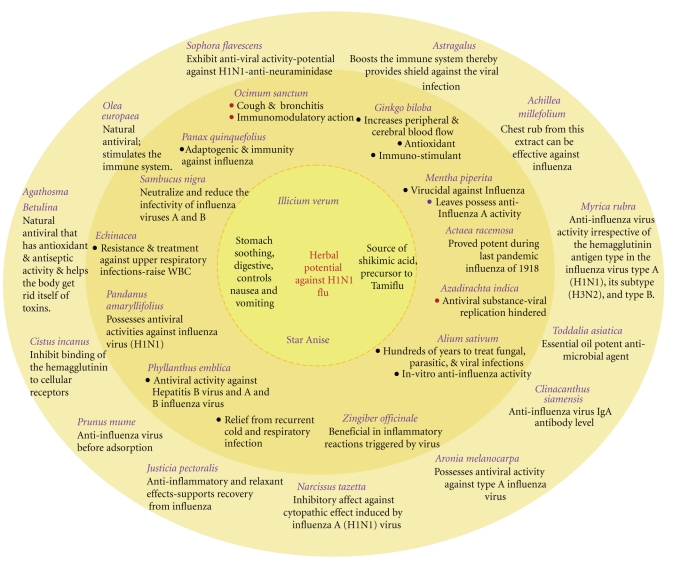
Medicinal plants present a plethora of options for the prevention and treatment of novel H1N1 in view of their multifarious modes of action.

Figure 7 summarizes some of the medicinal plants.

## 8. Conclusion

Pandemic influenza has posed a global threat, though currently the situation is fairly under control. However, the society remains insufficiently equipped to handle the outbreak from resistant strains if they were to occur despite many years of advance warning and preparation to handle such casualties. Mainstream medicine currently does not have a vaccine. The vaccine when available would be stockpiled along with antiviral drugs for use to handle crisis emerging. However, immediately after the launch it would be in short supply and ineffective, in a pandemic situation, for treating the masses in developing and underdeveloped nations. Besides the development of drug resistance, emergence of mutant strains of the virus, emergence of a more virulent strain, prohibitive costs of available drugs, time lag between vaccine development, and mass casualties would pose really difficult problems. In view of this, complementary and alternative medicine offers a plethora of interesting possibilities to help patients. Herbs exhibit a diverse array of biological activities and can be effectively harnessed for managing pandemic flu. It is evident that nutritional and botanical approaches, taken together, provide very potent tools for controlling an array of viral infections. It is unfortunate that many of these herbs have been overlooked, and the initial exciting research findings have not been followed up with larger, more rigorous clinical trials. The availability of a wide range of potentially active herbs and constituents, to potentiate as anti-influenza agents, may have a leading role in the ongoing struggle against the novel H1N1 infection. Herbal drugs would be useful, but it should not be expected as a panacea for offering perfect protection or absolute cessation of symptoms, but could be instead helpful in achieving reduced risk, symptom reduction, and quicker recovery. This paper portrays an eclectic overview for the treatment of pandemic influenza and covers some of the herbs that are most likely to be of deal help in managing the current pandemic scenario and also to cope with the next pandemic that might appear in the not-too-distant future. It is our firm belief that the plants discussed in the paper would also be useful in treating the patients with serious influenza in nonpandemic situations too.

## Figures and Tables

**Table 1:** List of medicinal plants from all over the world, which may prove useful to combat Swine flu.

S. no.	Plant and its origin	Native	Family	Principal chemical compounds	Anti-influenza action	Reference
1	*Clinacanthus siamensis *	Thailand	Acanthaceae	Trans-3-methylsulfonyl-2-propenol, trans-3-methylsulfinyl-2-propenol	Significant production of anti-influenza virus IgG(1) antibody produced a higher anti-influenza virus IgA antibody level.	[38]

2	*Myrica rubra *	Japan	Myricaceae	Phenolic compounds, chlorogenic acid, Catechol, Gallic acid	Anti-influenza virus activity irrespective of the hemagglutinin antigen type in the influenza virus type A (H1N1), its subtype (H3N2), and type B.	[39]

3	*Narcissus tazetta *	China	Amaryllidaceae	A fetuin-binding peptide	Significantly inhibit the plaque formation by respiratory syncytial virus (RSV) and the cytopathic effect induced by influenza A (H1N1) virus.	[40]

4	*Prunus mume*	Japan	Rosaceae	Presence of a lectin-like molecule	Strong in vitro anti-influenza activity against human influenza A viruses before viral adsorption.	[41]

5	*Cistus incanus*	Mediterranean	Cistaceae	Polymeric polyphenol components	Inhibit binding of the hemagglutinin to cellular receptors thereby, a local application of the extract at the viral entry routes may be a promising approach and thus help to provide protection from influenza virus infections.	[42]

6	*Pandanus amaryllifolius*	Malaysia, Indonesia	Pandanaceae	A Lectin called Pandanin	Possesses antiviral activities against influenza virus (H1N1).	[43]

8	*Sophora flavescens*	Korea	Leguminosae	Pterocarpans and flavanones	Ability to inhibit neuraminidase an enzyme identified as crucial for the proliferation of the influenza virus.	[44]

9	*Aronia melanocarpa*	North America	Rosaceae	Phenolic substances anthocyanins, glycosides	Antiviral activity against type A influenza virus.	[45]

10	*Schefflera heptaphylla*	China	Araliaceae	Frodin and Saponin	Potent antiviral activity against respiratory syncytial virus.	[46]

11	*Bergenia ligulata*	Nepal	Saxifragaceae	Condensed Tannins	Inhibits viral RNA synthesis and reduces viral peptide synthesis.	[47]

12	*Geranium sanguineum*	Bulgaria	Geraniaceae	Polyphenols	Inhibition of influenza virus protein synthesis.	[48]

13	*Sambucus nigra*	Japan	Caprifoliaceae	Kaempferol, astragalin, quercetin, rutin, isoquercitrin, hyperoside	Neutralize and reduce the infectivity of influenza viruses A and B.	[49]

14	*Justicia pectoralis*	Latin America	Acanthaceae	Coumarin and umbelliferone	Possess anti-inflammatory and relaxant effects, and it supports recovery from influenza.	[50]

15	*Achillea millefolium*	N. hemisphere	Asteraceae	Isovaleric acid, salicylic acid, asparagin, sterols, flavonoids, bitters, tannins, and coumarins	A chest rub from this extract can be effective against influenza.	[51]

16	*Astragalus*	Temperate region of N. hemisphere.	Fabaceae	Cycloastregenol	Boosts the immune system thereby provides shield against the Viral infection.	[52]

17	*Agathosma betulina*	Western-South Africa.	Rutaceae	Isomenthone and diosphenol	Natural antiviral that has antioxidant & antiseptic activity & helps the body get rid itself of toxins.	[53]

19	*Olea europaea*	Mediterranean Basin	Oleaceae	Phenolic compounds and oleuropein	Natural antiviral; stimulates the immune system.	[54]

20	*Junipers*	Arctic region, tropical Africa, central America	Cupressaceae	*α*-pinene, camphene, *β*-pinene, sabinene, myrcene, *α*-phellandrene, *α*-terpinene	It has high Macrophage immunomodulatory efficacies.	[55]

21	*Cinnamomum verum*	Sri Lanka	Lauraceae	Cinnamaldehyde, gum, tannin, mannitol	Antioxidants and radical scavenger. Prevents the entry of Virus.	[56]

22	*Scutellaria baicalensis*	Japan	Lamiaceae	5,7,4′-trihydroxy-8-methoxyflavone (F36) from the roots	Reduces the replication of influenza virus A/PR/8/34 (A/PR8) by inhibiting the fusion of the virus with endosome/lysosome membrane which occurs at early stage of viral infection cycle.	[57]

23	*Chrysanthemum indicum*	India, China	Asteraceae	*α*-terpineol, cis-sabinol, thujone, camphor	Chrysanthemum tea has many purported medicinal uses, including an aid in recovery from influenza.	[58]

**Table 2:** List of medicinal plants, from all over the world, which may prove useful in combating novel H1N1 flu by stimulating immune system.

Plant and its origin	Native	Family	Principal chemical compounds	Anti-influenza action	Reference
*Quillaja saponaria*	Central Chile	Quillajaceae	Glucoside saponin, tannins, polyphenols, calcium oxalate	Facilitate the removal of phlegm through coughing, hence aids in passive immunity.	[65]

*Echinacea purpurea*	North America	Compositae	Cichoric acid, ethanol- and ethyl acetate	Echinacea increases the levels of a chemical called properdin in the body which activates part of the immune system responsible for increasing the defence mechanisms against bacteria and viruses. It is also a potent anti-viral photosensitizer.	[66]

*Salvia officinalis*	Central and Southwestern Asia, Mexico	Lamiaceae	4-hydroxy-acetophenone-4-O-*β*-D-apiofuranosyl-(1→6)-O-*β*-D-glucopyranoside	Salviae extract increased the levels of influenza virus specific antibodies and neutralizing activities also acts as mucosal adjuvant and immune modulator during influenza virus infection.	[67]

*Nicotiana benthamiana*	Australia	Solanaceae	Capsanthin, Carotenoids, glycolipids.	Induce a protective immune response against a lethal influenza viral challenge in mice against the influenza.	[68]

*Ginkgo biloba*	China and Japan	Ginkgoaceae	Flavonoid glycosides and terpenoids (ginkgolides, bilobalides)	Ginkgo, improves blood flow and improves immunity.	[69]

*Thuja occidentalis*	North America, Europe	Cupressaceae	Thujone, Vitamin C	Leukocyte and cytokine induction, increases antibody response.	[70]

*Withania somnifera*	India	Solanaceae	Anaferine, anahygrine, beta-sisterol, chlorogenic acid, cysteine, cuscohygrine, pseudotropine, scopoletin, somniferinine, withaferin *α*, withanine, withananine, and withanolides	Stimulant for the immune system, also a very potent adaptogen.	[71]
